# Comparative evaluation of 19 reverse transcription loop-mediated isothermal amplification assays for detection of SARS-CoV-2

**DOI:** 10.1038/s41598-020-80314-0

**Published:** 2021-02-03

**Authors:** Yajuan Dong, Xiuming Wu, Shenwei Li, Renfei Lu, Yingxue Li, Zhenzhou Wan, Jianru Qin, Guoying Yu, Xia Jin, Chiyu Zhang

**Affiliations:** 1 Shanghai Public Health Clinical Center, Fudan University, Shanghai, 201508 China; 2 College of Life Sciences, Henan Normal University, Xinxiang, 453007 China; 3 Pathogen Discovery and Evolution Unit, Institut Pasteur of Shanghai, Chinese Academy of Sciences, Shanghai, China; 4 Shanghai International Travel Healthcare Center, Shanghai, 200335 China; 5 Clinical Laboratory, Nantong Third Hospital Affiliated To Nantong University, Nantong, 226006 China; 6 CAS Key Laboratory of Bio-Medical Diagnostics, Suzhou Institute of Biomedical Engineering and Technology, Chinese Academy of Sciences, Suzhou, 215163 China; 7 Medical Laboratory of Taizhou Fourth People’s Hospital, Taizhou, 225300 China; 8 Present Address: Tobacco Research Institute, Chinese Academy of Agricultural Sciences, Qingdao, Shandong 266101 China

**Keywords:** Microbiology, Molecular biology

## Abstract

Coronavirus disease 2019 (COVID-19) caused by SARS-CoV-2 has caused a global pandemics. To facilitate the detection of SARS-CoV-2 infection, various RT-LAMP assays using 19 sets of primers had been developed, but never been compared. We performed comparative evaluation of the 19 sets of primers using 4 RNA standards and 29 clinical samples from COVID-19 patients. Six of 15 sets of primers were firstly identified to have faster amplification when tested with four RNA standards, and were further subjected to parallel comparison with the remaining four primer sets using 29 clinical samples. Among these 10 primer sets, Set-4 had the highest positive detection rate of SARS-CoV-2 (82.8%), followed by Set-10, Set-11, and Set-13 and Set-17 (75.9%). Set-14 showed the fastest amplification speed (Tt value < 8.5 min), followed by Set-17 (Tt value < 12.5 min). Based on the overall detection performance, Set-4, Set-10, Set-11, Set-13, Set-14 and Set-17 that target *Nsp3*, *S, S, E*, *N* and *N* gene regions of SARS-CoV-2, respectively, were determined to be better than the other primer sets. Two RT-LAMP assays with the Set-4 primers in combination with any one of four other primer sets (Set-14, Set-10, Set-11, and Set-13) were recommended to be used in the COVID-19 surveillance.

## Introduction

Coronavirus disease 2019 (COVID-19), caused by the newly discovered coronavirus SARS-CoV-2^1, 2^, is rapidly spreading throughout the world, posing a huge challenge to global public health security. As of 20 September, 2020, it has infected over 30.6 million people, and resulted in at least 950,000 deaths globally. In the absence of effective antiviral drugs or efficacious vaccines, early diagnosis of SARS-CoV-2 infection is essential for the containment of COVID-19^3,4^, without which it is impossible to timely implement intervention and quarantine measures, and difficult to track contacts in order to limit virus spread.

Nucleic acid testing of various approaches are widely used as the primary tool for diagnosing COVID-19^3,4^. Among them, reverse transcription quantitative PCR (RT-qPCR) methods have been set as the gold standard for laboratory confirmation of SARS-CoV-2 infection because of their proven track record as being the most robust technology in molecular diagnostics^4–6^. However, the RT-qPCR assay relies on sophisticated facilities with reliable supply of electricity and well-trained personnel in large general hospitals and health care facilities, or government labs (such as CDC), and it is relatively time-consuming (about 1.5–2 h). These limit its capacity in point-of-care settings. Moreover, visiting a clinical setting for testing increases the risk of spreading the virus. Therefore, an alternative, fast, simple, and sensitive point-of-care testing (POCT) is highly needed to facilitate the detection of SARS-CoV-2 infection in resource-limited settings^3,7^.

Loop-mediated isothermal amplification (LAMP) is a promising POCT method with high sensitivity, specificity, and rapidity, and it is easy-to-use^8^. To overcome the limitation of RT-qPCR assay, a number of RT-LAMP assays using at least 19 sets of different primers had been developed in the last few months for the detection of SARS-CoV-2^9–19^. Although these assays had proven sensitive and effective for the detection of SARS-CoV-2, how do they compare with each other have not been evaluated. In this study, we compared all 19 sets of SARS-CoV-2-specific RT-LAMP primers using the mismatch-tolerant LAMP system that is faster and more sensitive than the conventional ones^20,21^, and screened the high-efficiency RT-LAMP assays for use in the detection of SARS-CoV-2.

## Results

### Strategy for the comparative evaluation

**Figure 1:**
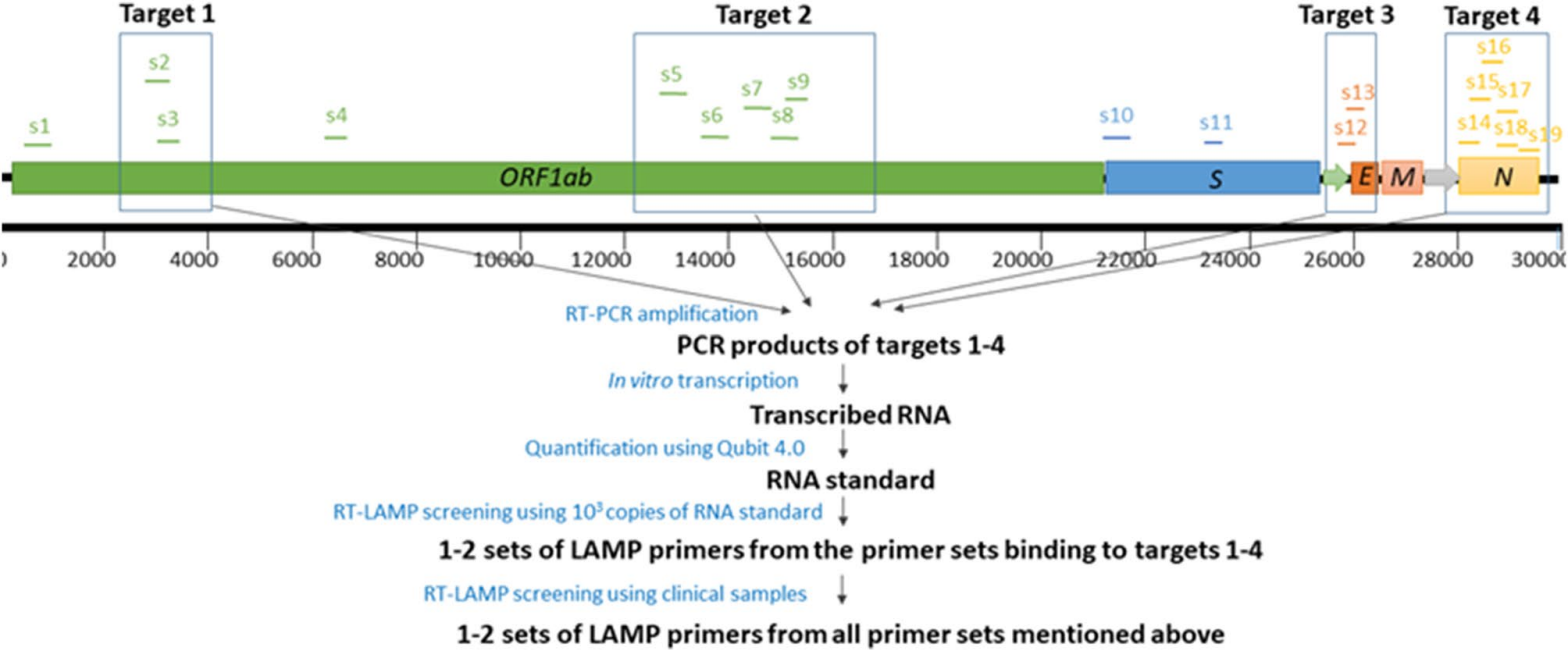
Genome location and evaluation strategy of 19 sets of SARS-CoV-2 RT-LAMP primers. The location of each primer set was detailed in Table 1.

There were 19 sets of SARS-CoV-2 RT-LAMP primers available for the evaluation on 6 April, 2020^9–19^. Among these primers, 2 sets were designed for binding to *Nsp3* (non-structural proteins), 5 for *RdRp* (RNA-dependent RNA polymerase), 2 for *E* (envelope protein) and 6 for *N* (nucleocapsid protein) gene regions of SARS-CoV-2 (Fig. 1). These regions are highly conserved among SARS-CoV-2 and SARS-CoV, but distinct from five other human coronaviruses (MERS-CoV, OC43, 229E, NL63 and HKU1). Other 4 sets of primers were dispersed throughout the genome of SARS-CoV-2, and are located in the genomic regions of leader protein (Set-1), *Nsp3* (Set-4), and *S* (spike protein) (Set-10 and Set-11) genes. Two to six sets of primers are adjacent to each other in the genomic location and 15 sets target to four genomic regions with lengths of 251–1954 bps. To minimize the consumption of clinical samples, and economize experimental efforts, we adopted a strategy that initiated by a preliminary evaluation of the primers binding to the four major genomic regions using in vitro-transcribed RNA standard, and followed by a further evaluation using clinical RNA samples (Fig. 1). Because four primer sets (i.e. S1, S4, S10 and S11) are dispersed throughout the genome (1–24,000 nt) and are not close to each other on the genome, it is difficult to obtain a long RNA template (about 24,000 nt length) covering the four primer sets by in vitro transcription (Fig. 1). Therefore, we directly moved the four primer sets to the next round of comparative experiments using clinical samples together with preliminarily selected primers.

### Preliminary evaluation of primer sets

**Figure 2:**
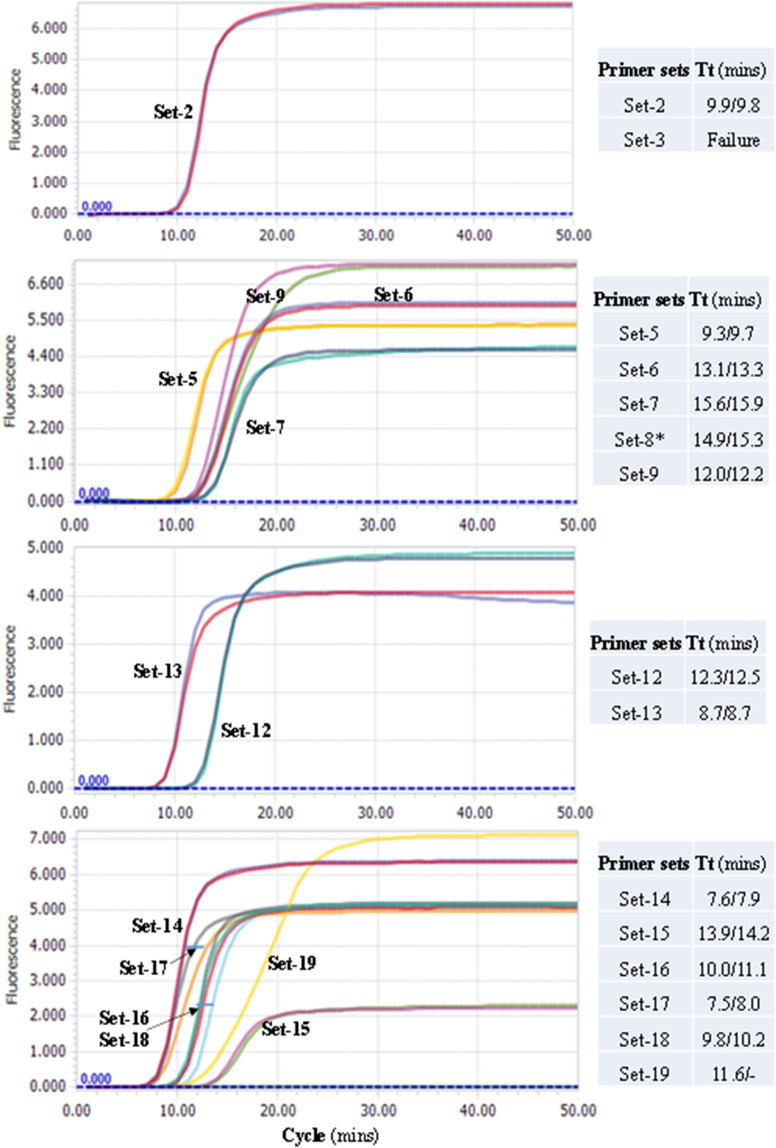
Comparison of performance of 15 RT-LAMP assays using RNA standards. Two replicates were performed for each primer set. The replicates of the same primer set often generated completely overlapped amplification curves. A short bar was used to highlight the non-overlapping curves of the same primer. The curves of non-template control (NTC) are not shown. *The Tt values of the Set-8 were obtained by another repeated comparative experiments with Set-5and Set-9, both of which showed a consistent trend, but slightly lower Tt values than those shown here.

Using 3000 copies of in vitro-transcribed RNA standards of four gene segments (Targets 1–4) of SARS-CoV-2 (Fig. 1), we assessed the amplification performance of 15 sets of RT-LAMP primers. Except for Set-3 that failed in amplification, all other primer sets generated amplification curves with Time threshold (Tt) of 7.5–15.9 min and reached the plateau phase within 20 min (Fig. 2). In particular, six sets of the primers showed faster amplification with 10 min less Tt values than other primer sets (Fig. 2). The six sets of primers contain three (Set-14, Set-17 and Set-18) that bind to *N* gene and another three (Set-2, Set-5 and Set-13) that bind to *Nsp*, *RdRp*, and *E* genes, respectively*.* Faster amplification is often associated with higher detection sensitivity^20^. The six sets of primers were selected for further evaluation using clinical samples together with other four primer sets that bind to other genomic regions of the virus.

### Comparative evaluation of ten primer sets using clinical samples

**Figure 3:**
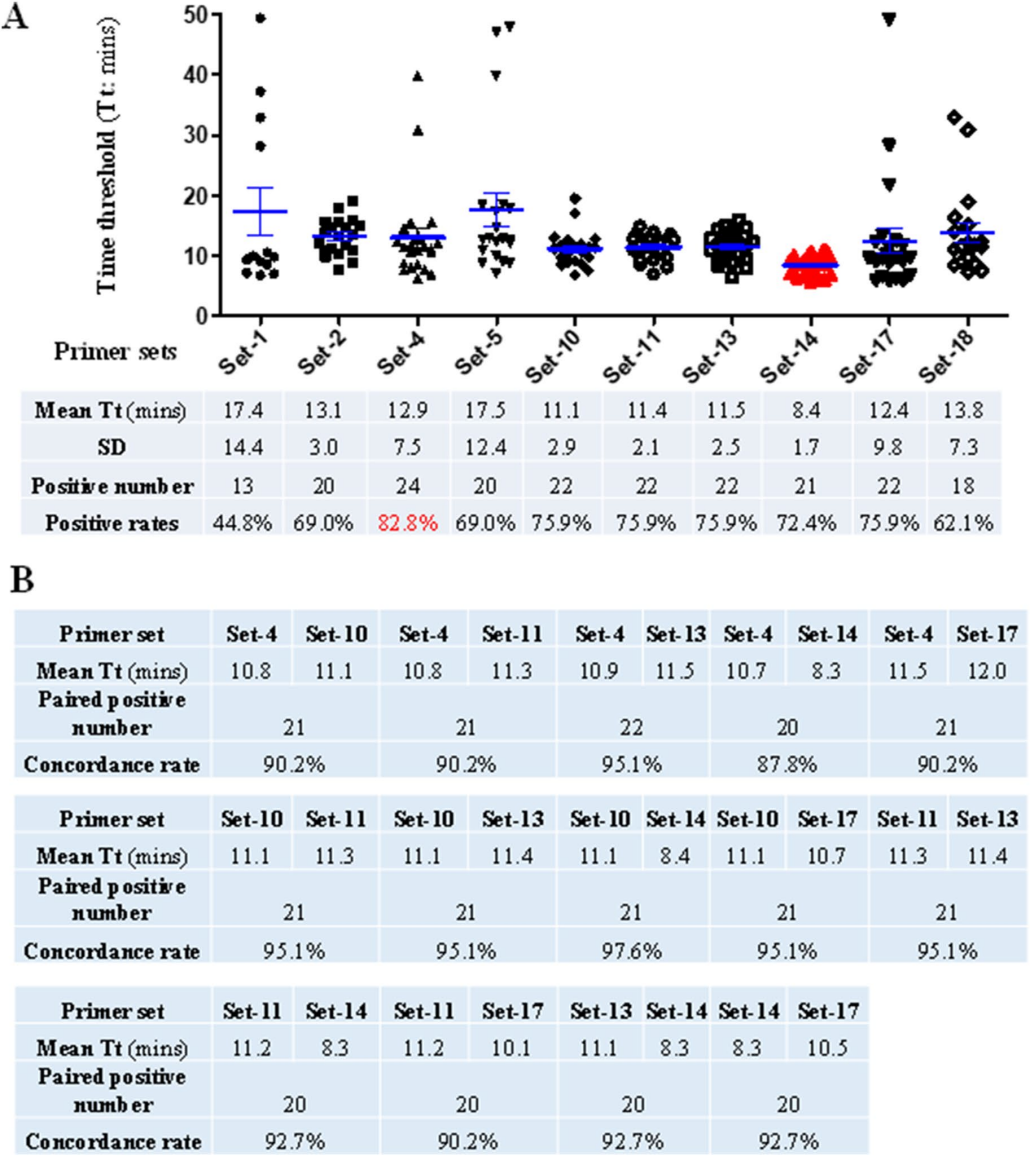
Comparison of performance of 10 selected RT-LAMP primer sets using 41 clinical RNA samples. (**A**) Positive rates and Tt values of 10 selected RT-LAMP assays. The 41 clinical samples included 29 SARS-CoV-2 positive and 12 negative clinical samples that were previously determined by RT-qPCR assay^28^. The positive rate was calculated by dividing the number of positive sample by each primer set by total positive sample number of RT-qPCR assay (i.e. 29). (**B**) Paired comparison of Tt values of the primers Set-4, Set-10, Set-11, Set-13, Set-14 and Set-17. Because all RNA from clinical samples were fourfold diluted and some of them have very low viral load (high Ct values by RT-qPCR assay), some positive samples were not detected as positive by the RT-LAMP assay, which are defined as false-negative. We calculated the concordance rate by dividing the number of consistent results (true positive, true negative and false-negative) by any two primer sets by the total sample number (i.e. 41). *SD* standard deviation.

A total of 29 RNA samples extracted from clinical samples of COVID-19 patients were used at fourfold dilutions. Except one sample, all 29 RNA samples were detected as SARS-CoV-2 positive by at least one of the primer sets. Nine samples were detected as positive by all ten sets of primers and almost all reactions (except one with 49.5 min) had Tt values of less than 15.1 min, indicating high viral load. The primer Set-4 detected 24 positive samples, showing the highest positive detection rate (82.8%), followed by Set-10, Set-11, Set-13 and Set-17 that all detected 22 positive samples (75.9%) (Fig. 3A). Two primer sets, Set-1 and Set-18, had the lowest positive detection rates of 44.8% and 62.1%, respectively, and thus were excluded in the subsequent analyses. Comparison showed that the primer Set-14 had the lowest mean Tt value of less than 8.4 min, followed by Set-10, Set-11 and Set-13 that had mean Tt values of 11.1–11.5 min (Fig. 3A). These four fast-amplification sets of primers also had small standard deviations (SD) of 1.7–2.9, indicating that the RT-LAMP with these four primer sets were relatively more stable and faster than the other 15 primer sets. Compared with other primer sets, the Set-14 was the most efficient one that generated the fastest (the lowest Tt value) and the second fastest amplification in 14 and 7 samples, respectively, followed by Set-17 which was the fastest in 6 samples and second best in 9 samples, demonstrating these two primer sets had the best performance.

**Figure 4:**
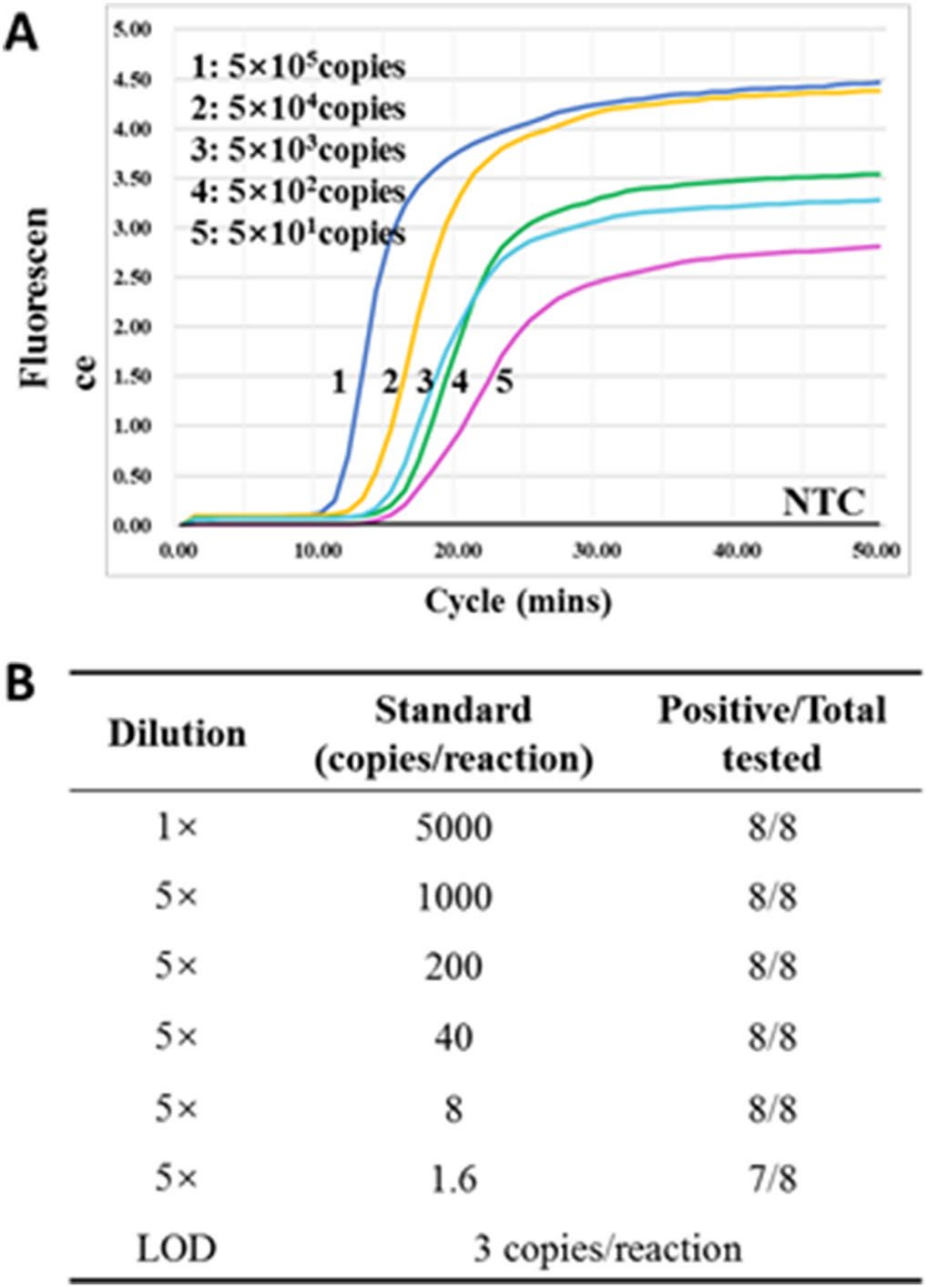
Sensitivity (**A**) and LOD (**B**) of the primer Set-4. *NTC* non-template control.

Because of their relatively high positive detection rates and lower Tt values, six primer sets including Set-4, Set-10, Set-11, Set-13, Set-14 and Set-17 were subjected to further pairwise comparison. The comparison showed that any two sets of these primers had high concordance performance (87.8–97.6%) for 41 clinical RNA samples (including 29 positive and 12 negative for SARS-CoV-2) (Fig. 3B). All the six primer sets had high amplification efficiency with mean Tt values of less than 12 min (Fig. 3B). Because of the highest positive detection rate, we further tested the sensitivity of the primer set-4. The results showed that it had the limit of the detection (LOD) of 3 copies per 25 µL reaction (Fig. 4), indicating a higher sensitivity than previously reported (Table 1)^14^.

**Table 1:** Information of 19 sets of RT-LAMP primers for the detection of SARS-CoV-2.

Primer sets	Primer name	Primer sequence (5′-3′)	Target gene	Genomic location (nt)	Sensitivity (or LOD)	Refs
S1	F3	CTGCACCTCATGGTCATGTT	*orf1ab*	498–711	1200 copies/25 μL reaction	^18^
	B3	AGCTCGTCGCCTAAGTCAA				
	FIP	GAGGGACAAGGACACCAAGTGTATGGTTGAGCTGGTAGCAGA				
	BIP	CCAGTGGCTTACCGCAAGGTTTTAGATCGGCGCCGTAAC				
	LF	CCGTACTGAATGCCTTCGAGT				
	LB	TTCGTAAGAACGGTAATAAAGGAGC				
S2	F3	TCCAGATGAGGATGAAGAAGA	*orf1ab*	3043–3331	1.02 fg/25 μL reaction	^19^
	B3	AGTCTGAACAACTGGTGTAAG				
	FIP	AGAGCAGCAGAAGTGGCACAGGTGATTGTGAAGAAGAAGAG				
	BIP	TCAACCTGAAGAAGAGCAAGAACTGATTGTCCTCACTGCC				
	LF	CTCATATTGAGTTGATGGCTCA				
	LB	ACAAACTGTTGGTCAACAAGAC				
S3	F3	GGAATTTGGTGCCACTTC	*orf1ab*	3145–3345	100 copies/15 μL reaction	^14^
	B3	CTATTCACTTCAATAGTCTGAACA				
	FIP	CTTGTTGACCAACAGTTTGTTGACTTCAACCTGAAGAAGAGCAA				
	BIP	CGGCAGTGAGGACAATCAGACACTGGTGTAAGTTCCATCTC				
	LF	ATCATCATCTAACCAATCTTCTTC				
	LB	TCAAACAATTGTTGAGGTTCAACC				
S4	F3	TGCAACTAATAAAGCCACG	*orf1ab* (*Nsp3*)	6253–6446	100 copies/15 μL reaction	^14^
	B3	CGTCTTTCTGTATGGTAGGATT				
	FIP	TCTGACTTCAGTACATCAAACGAATAAATACCTGGTGTATACGTTGTC				
	BIP	GACGCGCAGGGAATGGATAATTCCACTACTTCTTCAGAGACT				
	LF	TGTTTCAACTGGTTTTGTGCTCCA				
	LB	TCTTGCCTGCGAAGATCTAAAAC				
S5	F3	TGCTTCAGTCAGCTGATG	*orf1ab*	13,434–13,636	7 copies/10 µL reaction	^13^
	B3	TTAAATTGTCATCTTCGTCCTT				
	FIP	TCAGTACTAGTGCCTGTGCCCACAATCGTTTTTAAACGGGT				
	BIP	TCGTATACAGGGCTTTTGACATCTA TCTTGGAAGCGACAACAA				
	LF	CTGCACTTACACCGCAA				
	LB	GTAGCTGGTTTTGCTAAATTCC				
S6	F3	GGTATGATTTTGTAGAAAACCCA	*orf1ab*	13,925–14,140	20 copies/25 µL reaction	^12^
	B3	CAACAGGAACTCCACTACC				
	FIP	GGCATCACAGAATTGTACTGTTTTTGCGTATACGCCAACTTAGG				
	BIP	AATGCTGGTATTGTTGGTGTACTGAGGTTTGTATGAAATCACCGAA				
	LF	AACAAAGCTTGGCGTACACGTTCA				
S7	F3	GTTACGATGGTGGCTGTA	*orf1ab*	14,885–15,081	5 copies/25 µL reaction	^16^
	B3	GGCATACTTAAGATTCATTTGAG				
	FIP	AGCCTTACCCCATTTATTAAATGGAGCTAACCAAGTCATCGTCAA				
	BIP	AATGAGTTATGAGGATCAAGATGCATTATAGTAGGGATGACATTACGT				
	LF	AAACCAGCTGATTTGTCTAGGTTG				
S8	F3	AAACGTAATGTCATCCCTACT	*orf1ab* (*RdRp*)	15,034–15,274	3 copies/25 μL reaction	^9^
	B3	GGTTTTCTACATCACTATAAACAGT				
	FIP	ACAGATAGAGACACCAGCTACGCTCAAATGAATCTTAAGTATGCCA				
	BIP	ATAGCCGCCACTAGAGGAGCCCAACCACCATAGAATTTGC				
	LF	GTGCGAGCTCTATTCTTTGCACTA				
S9	F3	CCACTAGAGGAGCTACTGTA	*orf1ab*	15,182–15,387	10 copies/20 µL reaction	^11^
	B3	TGACAAGCTACAACACGT				
	FIP	AGGTGAGGGTTTTCTACATCACTATATTGGAACAAGCAAATTCTATGG				
	BIP	ATGGGTTGGGATTATCCTAAATGTGTGCGAGCAAGAACAAGTG				
	LF	CAGTTTTTAACATGTTGTGCCAACC				
	LB	TAGAGCCATGCCTAACATGCT				
S10	F3	CTGACAAAGTTTTCAGATCCTCAG	*S*	21,678–21,886	NA	^14^
	B3	AGTACCAAAAATCCAGCCTCTT				
	FIP	TCCCAGAGACATGTATAGCATGGAATCAACTCAGGACTTGTTCTTACC				
	BIP	TGGTACTAAGAGGTTTGATAACCCTGTTAGACTTCTCAGTGGAAGCA				
	LF	CCAAGTAACATTGGAAAAGAAA				
	LB	GTCCTACCATTTAATGATGGTGTTT				
S11	F3	TCTATTGCCATACCCACAA	*S*	23,693–23,937	200 copies/25 µL reaction	^12^
	B3	GGTGTTTTGTAAATTTGTTTGAC				
	FIP	CATTCAGTTGAATCACCACAAATGTGTGTTACCACAGAAATTCTACC				
	BIP	GTTGCAATATGGCAGTTTTTGTACATTGGGTGTTTTTGTCTTGTT				
	LF	ACTGATGTCTTGGTCATAGACACT				
	LB	TAAACCGTGCTTTAACTGGAATAGC				
S12	F3	CCGACGACGACTACTAGC	*E*	26,191–26,424	20 copies/10 µL reaction	^15^
	B3	AGAGTAAACGTAAAAAGAAGGTT				
	FIP	CTAGCCATCCTTACTGCGCTACTCACGTTAACAATATTGCA				
	BIP	ACCTGTCTCTTCCGAAACGAATTTGTAAGCACAAGCTGATG				
	LF	TCGATTGTGTGCGTACTGC				
	LB	TGAGTACATAAGTTCGTAC				
S13	F3	AGCTGATGAGTACGAACTT	*E*	26,226–26,441	5 copies /25 μL reaction	^16^
	B3	TTCAGATTTTTAACACGAGAGT				
	FIP	ACCACGAAAGCAAGAAAAAGAAGTATTCGTTTCGGAAGAGACAG				
	BIP	TTGCTAGTTACACTAGCCATCCTTAGGTTTTACAAGACTCACGT				
	LB	CTGCGCTTCGATTGTGTGCGT				
S14	F3	CCAGAATGGAGAACGCAGTG	*N*	28,354–28,569	1 copies/25 µL reaction	^17^
	B3	CCGTCACCACCACGAATT				
	FIP	AGCGGTGAACCAAGACGCAGGGCGCGATCAAAACAACG				
	BIP	AATTCCCTCGAGGACAAGGCGAGCTCTTCGGTAGTAGCCAA				
	LF	TTATTGGGTAAACCTTGGGGC				
	LB	TTCCAATTAACACCAATAGCAGTCC				
S15	F3	TGGCTACTACCGAAGAGCT	*N*	28,525–28,741	120 copies/25 μL reaction	^18^
	B3	TGCAGCATTGTTAGCAGGAT				
	FIP	TCTGGCCCAGTTCCTAGGTAGTCCAGACGAATTCGTGGTGG				
	BIP	AGACGGCATCATATGGGTTGCACGGGTGCCAATGTGATCT				
	LF	GGACTGAGATCTTTCATTTTACCGT				
	LB	ACTGAGGGAGCCTTGAATACA				
S16	F3	AGATCACATTGGCACCCG	*N*	28,702–28,914	5 copies/25 µL reaction	^16^
	B3	CCATTGCCAGCCATTCTAGC				
	FIP	TGCTCCCTTCTGCGTAGAAGCCAATGCTGCAATCGTGCTAC				
	BIP	GGCGGCAGTCAAGCCTCTTCCCTACTGCTGCCTGGAGTT				
	LF	GCAATGTTGTTCCTTGAGGAAGTT				
	LB	CGTAGTCGCAACAGTTAAGAAATTC				
S17	F3	GCCAAAAGGCTTCTACGCA	*N*	28,774–28,971	NA	^14^
	B3	TTGCTCTCAAGCTGGTTCAA				
	FIP	TCCCCTACTGCTGCCTGGAGGCAGTCAAGCCTCTTCTCG				
	BIP	TCTCCTGCTAGAATGGCTGGCATCTGTCAAGCAGCAGCAAAG				
	LF	TGTTGCGACTACGTGATGAGGA				
	LB	ATGGCGGTGATGCTGCTCT				
S18	F3	GCCAAAAGGCTTCTACGCA	*N*	28,774–28,971	20 copies /25 µL reaction (118.6 copies/25 µL reaction)	^10^
	B3	TTGCTCTCAAGCTGGTTCAA				
	FIP	TCCCCTACTGCTGCCTGGAGCAGTCAAGCCTCTTCTCGTT				
	BIP	TCTCCTGCTAGAATGGCTGGCATCTGTCAAGCAGCAGCAAAG				
	LB	TGGCGGTGATGCTGCTCTT				
S19	F3	AACACAAGCTTTCGGCAG	*N*	29,083–29,311	20 copies/10 µL reaction	^15^
	B3	GAAATTTGGATCTTTGTCATCC				
	FIP	CGCATTGGCATGGAAGTCACTTTGATGGCACCTGTGTAG				
	BIP	TGCGGCCAATGTTTGTAATCAGCCAAGGAAATTTTGGGGAC				
	LF	TTCCTTGTCTGATTAGTTC				
	LB	ACCTTCGGGAACGTGGTT				

F3/B3: outer primers; FIP/BIP: forward and backward internal primers; LF/LB: forward and backward loop primers.

### Specficity evaluation of six optimal primer sets based on sequence alignment

The specificity of these primer sets had been reported in previous studies^9–19^. In previous specificity experiments, common human respiratory pathogens were used, and none amplification curve or very weak amplification signals were observed. The pathogens used in the specificity experiment of the selected primer sets in the previous papers are listed in supplementary Table S2. To further examine the specificity of six recommended primer sets (Set-4, Set-10, Set-11, Set-13, Set-14 and Set-17) to other human coronaviruses, we performed sequence alignment analyses. SARS-CoV-2 shares 79.5% genomic homology with SARS-CoV^1,2^, indicating a relatively high sequence identity; but it is largely distinct from MERS-CoV and other four human coronaviruses (Supplementary Fig. S1). In particular, several primers of Set-4, Set-10 and Set-17 correspond to gaps or insertions of the genomes of MERS-CoV and other four common human coronaviruses OC43, 229E, NL63 and HKU1. These results implied that these six sets of primers were unable to bind to the genomes of MERS-CoV and four common human coronaviruses, therefore more specific for SARS-CoV-2. However, because of high sequence identity and the use of mismatch-tolerant RT-LAMP system that allows the presence of few mismatched bases between primers and templates, the SARS-CoV-2 RT-LAMP assays may generate a cross-amplification of SARS-CoV. In addition, the six primer sets did not generate amplification for all 12 COVID-19 negative RNA samples within 50 min, indicating that there was not or less non-specific amplification.

## Discussion

SARS-CoV-2 transmission mainly occurs in the early and progressive stages of COVID-19 disease during which the patients and virus carriers have higher viral load than that in recovery stage^22–24^, and are generally more infectious. To contain the spread of the virus, early diagnosis is essential^3,4^. It helps to trigger timely intervention (e.g. quarantine, lockdown, and contact tracing), and facilitates to optimize clinical management. It is clear that serological assays are not suitable for this purpose, because detectable antibodies always appear several days after infection. Therefore, viral RNA testing is the primary method for early diagnosis of COVID-19. Despite being the most robust diagnostic tests, RT-qPCR-based assays are more centralized in core facilities, and they are not amenable for large-scale monitoring for asymptomatic and pre-symptomatic virus carriers in point-of-care settings (e.g. community and home). Therefore, community- and/or home-based nucleic acid assays that allow individuals to test in the community, at home, or other point-of-care sites without having to visit hospitals are convenient tools for the detection of SARS-CoV-2 infection by the general public^3,7^.

RT-LAMP assays are such needed tools^8,20,21^. In fact, various LAMP assays have been developed that included at least 19 sets of primers targeting to different genomic regions of SARS-CoV-2, with reported high detection sensitivity ranging from 1 to 1200 copies per 25 µL reaction^9–19^. However, these primers are never formally evaluated with clinical samples. The sensitivity and performance of a RT-LAMP assay are mainly determined by the primers, because other components of the reaction system are optimized and stable. Therefore, assessing the optimal RT-LAMP primer sets for the detection of SARS-CoV-2 infection is important for the selection of the best assay format to use for large field screening of COVID-19 patients.

Recently, the reaction system of RT-LAMP was further optimized to have higher sensitivity and faster amplification speed, even allowing the presence of few mismatched bases between primer and templates in a mismatch-tolerant version^20,21^. The new optimized reaction system containing an additional 0.15 U of high-fidelity DNA polymerase is called as mismatch-tolerant LAMP. The inclusion of an additional amount of high-fidelity DNA polymerase makes it a higher applicability to highly variable viruses, and a 10–15 min faster reaction speed than the conventional LAMP method. Using this new version, we assessed 19 sets of SARS-CoV-2 RT-LAMP primers. Six sets of primers showing faster amplification speed were firstly selected from 15 sets of primers using 4 RNA standards, and then tested with other 4 primer sets using 41 clinical samples. Eight sets of primers showed either comparable or better performance than the other 2 sets of primers (Set-1 and Set-18) as determined by positive detection rate. Of the 8 sets of primers, six were further selected based on high positive detection rate and/or overall faster amplification speed (with mean Tt value less than 13 min). The six primer sets are Set-4, Set-10, Set-11, Set-13, Set-14 and Set-17 that correspond to *Nsp3*, *S, S, E*, *N*, and *N* genes of SARS-CoV-2, respectively.

Among selected assays, the *N* gene-based RT-LAMP assays (Set-14 and Set-17) had the fastest amplification speed, followed by *Orf, S* and *E* gene-based assay (Set-4, Set-10, Set-11 and Set-13). This result suggested that the *N* gene-based RT-LAMP assay was more sensitive in detecting SARS-CoV-2 than that based on other genes, consisting with results of RT-qPCR assays^5^. In this study, Set-4 had the highest positive detection rates than all other primer sets, and had a LOD of 3 copies per 25 µL reaction, obviously more sensitive than the previously reported sensitivity of more than 100 copies per 25 µL reaction (Table 1 and Fig. 4)^12,14^. The sensitivity of Set-4 was comparable with highly sensitive primer sets Set-13 and Set-14 (less than 3 copies per 25 µL reaction)^16,17^. In addition, the sensitivity of primer Set-11 was less than 50 copies per 25 µL reaction (data not shown), obviously low 200 copies/25 µL reaction reported in previous study^12^. These indicated the mismatch-tolerant method significantly improved the detection sensitivity of RT-LAMP^20^. In addition, two of our previously reported primers, Set-8 and Set-18, exhibited high sensitivities of 3–20 copies per 25 µL reaction and good performance in the detection of clinical samples under the mismatch-tolerant reaction condition^9,10^, but they did not show better performance than other nine primer sets in this study. A reason might be that the use of the mismatch-tolerant reaction system generally improved the amplification efficiency of the primers reported by other groups^20^.

The analyzed primer sets showed high specificity in that they did not amplify any SARS-CoV-2 negative clinical samples. Sequence alignment analyses further supported that the six sets of optimal primers had good specificity to SARS-CoV-2, albeit they might generate non-specific amplification for SARS-CoV due to a high degree of sequence identity. However, given the lethal nature of both SARS-CoV-2 and SARS-CoV^25^, a non-specific positive result for SARS-CoV might also be of clinical importance.

Two nucleic acid assays targeting to different genes are suggested to be used in the detection of SARS-CoV-2 to avoid potential false-negative results^5^. Based on comparable performances, any two of the six optimal primer sets (Set-4, Set-10, Set-11, Set-13, Set-14 and Set-17) were recommend to be used in the detection of SARS-CoV-2. However, simultaneous use of Set-10 and Set-11, or Set-14 and Set-17 should be avoided because the former two sets target to the same *S* gene and the latter two sets target to the same *N* gene. In addition, because of highest positive detection rate and high sensitivity, Set-4 was strongly encouraged to be preferentially selected for the diagnosis of COVID-19 patients. Apart from the six recommend primer sets, other primers such as Set-2 and Set-5 also had good performance, and can also be used in the monitoring of COVID-19 infections.

Another advantage of our version of the RT-LAMP assay is that the results are easily visualized with a pH-sensitive indicator dye (e.g. cresol red and neutral red)^26^. Moreover, a combination of a nucleic acid extraction-free protocol and a master RT-LAMP mix containing all reagents (enzymes, primers, magnesium, nucleotides, dye and additives), except the template, enables the development of a simple kit that can be used at home, or a community-based diagnosis center for the detection of COVID-19 infection^3,27^.

In summary, we evaluated and selected six optimal primer sets from 19 sets of SARS-CoV-2 RT-LAMP primers through a comparative evaluation with clinical RNA samples from COVID-19 patients. Two RT-LAMP assays with the Set-4 primers and any one of the other four primer sets (Set-10, Set-11, Set-13 and Set-14) were recommended to be used in the COVID-19 surveillance to facilitate the early finding of asymptomatic and pre-symptomatic virus carriers in clinical and point-of-care settings, and the monitoring of environmental samples in the field.

## Materials and methods

### Ethics statement

The study was approved by Nantong Third Hospital Ethics Committee (E2020002: 3 February 2020). All experiments were performed in accordance with relevant guidelines and regulations. Written informed consents were obtained from each of the involved patients.

### Preparation of RNA standard

To prepare RNA standard, four SARS-CoV-2 genomic segments (2720–3620 nt, 13,403–15,502 nt, 25,901–26,700 nt and 28,274–29,533 nt in Wuhan-Hu-1, GenBank: MN908947.3) were amplified from previously confirmed positive RNA sample with T7-promoter-containing primers (Supplementary Table S1). RNA standard was generated by in vitro transcription^21^, and quantitated by Qubit 4.0 Fluorometer (Thermo Fisher Scientific, USA). Copy number of RNA standard was estimated using the formula: RNA copies/mL = [RNA concentration (g/μL)/(nt transcript length × 340)] × 6.022 × 10^23^.

### RNA samples of COVID-19 patients

A total of 29 RNA samples were obtained from COVID-19 patients described in our previous studies^9,10^. In brief, the throat swabs of COVID-19 patients were put into virus transport medium that contains Hank’s buffer, BSA, HEPES and antibiotics, and 300 μL were used for RNA extraction using RNA extraction Kit (Liferiver Biotechnology Co., Ltd., Shanghai) and eluted in 90 μL nuclease-free water. After screening and confirmation tests, the remaining RNA samples were stored at − 80 °C. When used for RT-LAMP assays, the stored SARS-CoV-2 positive RNA samples as confirmed by Novel Coronavirus (2019-nCoV) Real Time RT-PCR kit (Liferiver Biotechnology Co., Ltd., Shanghai) were thawed and fourfold diluted^28^. In addition, 12 SARS-CoV-2 negative clinical RNA samples were used as controls.

### RT-LAMP assay

To assess the performance of 19 sets of RT-LAMP primers in the detection of SARS-CoV-2, an optimized mismatch-tolerant RT-LAMP method that has higher sensitivity and faster amplification speed than the conventional ones was used. A 25 µL RT-LAMP reaction mixture was prepared with 1 × isothermal amplification buffer, 6 mM MgSO_4_, 1.4 mM dNTPs, 8 units of WarmStart Bst 2.0 DNA polymerase, 7.5 units of WarmStartR RT, 0.15 unit of Q5 High-Fidelity DNA Polymerase, 0.2 μM each of primers of F3 and B3, 1.6 μM each of primers of FIP and BIP, 0.4 μM each of loop primer LF and/or LB, and 0.4 mM SYTO 9 (Life technologies, Carlsbad, CA, United States). The enzymes were all purchased from New England Biolabs (Beverly, MA, United States). In general, 3 μL of RNA standard or samples were added into each RT-LAMP reaction. The reaction was run at 63 °C for 50 min with real-time monitoring by the LightCycler 96 real-time PCR System (Roche Diagnostics, Mannheim, Germany).

### Limit of detection (LOD)

Tenfold serial dilutions of the RNA standard were used as the standards to determine the sensitivity of selected primer sets. To test the LOD of the primer Set-4, fivefold serial dilutions of the RNA standard, from 5000 to 2 copies per 1 μL, were used. Each dilution was tested in a set of 8 replicates. A 95% probability of obtaining a positive result was used to define the LOD.

## Supplementary Information

Supplementary Information.

## Data Availability

All data was available from the article and author.
