# Supplementary material for: Comparative evaluation of 19 reverse transcription loop-mediated isothermal amplification assays for detection of SARS-CoV-2
Source: Sci Rep. 2021 Feb 3;11:2936. doi: 10.1038/s41598-020-80314-0 (PMC7858603; doi:10.1038/s41598-020-80314-0)
Supplement: Supplementary file 1 — Supplementary Information. [file 41598_2020_80314_MOESM1_ESM.docx]

**Supplementary Materials**

Comparative Evaluation of 19 Reverse Transcription Loop-Mediated Isothermal Amplification Assays for Detection of SARS-CoV-2

Yajuan Dong ^a, b ‡^, Xiuming Wu ^c ‡^, Shenwei Li ^d^, Renfei Lu ^e^, Yingxue Li^a,f^, Zhenzhou Wan ^g^, Jianru Qin ^b^, Guoying Yu ^b^, Xia Jin ^a^, Chiyu Zhang ^a *^

a Shanghai Public Health Clinical Center, Fudan University, Shanghai 201508, China;

b College of Life Sciences, Henan Normal University, Xinxiang 453007, China

c Pathogen Discovery and Evolution Unit, Institut Pasteur of Shanghai, Chinese Academy of Sciences, Shanghai 200031, China

d Shanghai International Travel Healthcare Center, Shanghai 200335, China

e Clinical Laboratory, Nantong Third Hospital Affiliated to Nantong University, Nantong 226006, China;

f  CAS key Laboratory of Bio-medical Diagnostics, Suzhou Institute of Biomedical Engineering and Technology, Chinese Academy of Sciences, Suzhou 215163, China

g Medical Laboratory of Taizhou Fourth People’s Hospital, Taizhou 225300, China;

^*^Corresponding author.

Prof. Chiyu Zhang, PhD, Shanghai Public Health Clinical Center, Fudan University, Shanghai 201508, China;, 2901 Caolang Road, Shanghai, China.

E-mail address: [zhangcy1999@](mailto:zhangcy1999@ips.ac.cn)shphc.org.cn

‡ Yajuan Dong and Xiuming Wu contributed equally to this work.

**Supplementary Table S1. Primers used for *in vitro* transcription.**

| Target 1 | Primer | Sequence (5’-3’) |
| --- | --- | --- |
| Target 1 (*Orf1ab*) | 1F-T7 | TAATACGACTCACTATAGGCACCAACAAAGGTTACTTT |
|  | 1R | CGACAACATGAAGACAGTGT |
| Target 2 (*Orf1ab*) | 2F-T7 | TAATACGACTCACTATAGGGCTGTAGTTGTGATCAACTC |
|  | 2R | CAGTTGTGGCATCTCCTGAT |
| Target 3 (*E*) | E-F-T7 | TAATACGACTCACTATAGCTTCAGGTGATGGCACAACA |
|  | E-R | CTGGCCATAACAGCCAGAG |
| Target 4 (*N*) | N-F-T7 | TAATACGACTCACTATAGATGTCTGATAATGGACCCCA |
|  | N-R | TTAGGCCTGAGTTGAGTCAG |

T7 promoter sequence is underlined.

**Supplementary Table S2. Pathogens used in the specificity experiments in previous studies.**

| Primer sets | Specificity assays |
| --- | --- |
| S1 | NA |
| S2 | MERS, BtCoV and MHV |
| S3 | hCoV-229E, hCoV-OC43 and MERS-CoV |
| S4* | HCoV-OC43 (VR-1558), HCoV-229E (VR-740) and MERS-CoV |
| S5 | lphacoronavirus (PEDV and TGEV); Gammacoronavirus (IBV); and Deltacoronavirus (PDCoV) |
| S6 | human coronavirus HCoV-229E, HCoV-NL63, HCoV-OC43, HCoV-HKU1, influenza A-H3N2, H1N1, influenza B, parainfluenza viruses (PIV) type 1/2/3/4, adenoviruses (ADV) type 1/2/3/4/5/6/7, respiratory syncytial virus (RSV) type A/B, human metapneumovirus (HMPV), human bocavirus (BoV), rhinovirus (Rh) type A/B/C, Mycoplasma pneumoniae (MP) strain M129/FH, Haemophilus influenza ATCC 49247, Staphylococcus aureus, Klebsiella pneumoniae, Streptococcus pneumoniae, and Pseudomonas aeruginosa |
| S7 | SARS-COV-2 negative samples, Flu A, Flu B, RSV |
| S8 | including influenza A, B, and C viruses, parainfluenza viruses type 1–3, enterovirus, respiratory syncytial virus (RSV) A and B groups, HCoV-HKU-1,  HCoV-NL63, human rhinovirus, human metapneumovirus, adenovirus and bocavirus , HCoV-OC43 (VR-1558) and HCoV-229E (VR-740) |
| S9 | sequence alignment：Bat-SL-Cov-HKU3-1、Bat-SL-CoV-Rf1、Bat-SL-CoV-ZC45、Bat-SL-CoV-Rp3、Bat-SL-CoV-ZXC21、HCoV-OC43、HCoV-HKU1、Influenza  A virus H3N2、Influenza B virus、SARS-CoV-Tor2、SARS-CoV-SZ3 |
| S10* | NA |
| S11* | HCoV-229E, HCoV-NL63, HCoV-OC43, HCoV-HKU1, H3N2, H1N1, influenza B, PIV 1-4, ADV 1-7, RSV A-B, HMPV 1-2, RH A-C, MP-FH, MP-M129; Haemophilus influenzae; Staphylococcus aureus; Klebsiella pneumoniae; Streptococcus pneumoniae; Pseudomonas aeruginosa |
| S12 | Influenza, OC43, HKU1, NL63 |
| S13* | SARS-CoV-2 negative samples, Flu A-B, RSV |
| S14* | HCoV-OC43 , HCoV-HKU1 , HCoV-229E , HCoV-NL63, Adenovirus, Respiratory syncytial virus A, Human parainfluenza 2 virus, Human parainfluenza 3 virus, H1N1 influenza virus, H5N1 influenza virus, H7N9 influenza virus, H9N2 influenza virus, Mycoplasma pneumoniae, Influenza B virus, Staphylococcus aureus, Mycobacterium tuberculosis, Legionella pneumophila, Candida albicans, Candida glabrata, Candida tropicalis, Aspergillus fumigatus, Cryptococcus neoformans |
| S15 | NA |
| S16 | SARS-CoV-2 negative samples, Flu A-B, RSV |
| S17* | HCoV-229E, HCoV-NL63, HCoV-OC43, HCoV-HKU1, H3N2, H1N1, influenza B, PIV 1-4, ADV 1-7, RSV A-B, HMPV 1-2, RH A-C, MP-FH, MP-M129; Haemophilus influenzae; Staphylococcus aureus; Klebsiella pneumoniae; Streptococcus pneumoniae; Pseudomonas aeruginosa |
| S18 | HCoV-229E, HCoV-NL63, HCoV-OC43, HCoV-HKU1, H3N2, H1N1, influenza B, PIV 1-4, ADV 1-7, RSV A-B, HMPV 1-2, RH A-C, MP-FH, MP-M129; Haemophilus influenzae; Staphylococcus aureus; Klebsiella pneumoniae; Streptococcus pneumoniae; Pseudomonas aeruginosa |
| S19 | Influenza, OC43, HKU1, NL63 |

The information was retrieved from Refs 9-19.

* Recommended primer sets in this study. NA, not available.

**Supplementary Table S3. Comparison of the qRT-PCR Ct values with mean Tt values of the RT-LAMP assays.**

| Sample No. | CT of RT-qPCR  （cycle number） | Mean Tt of RT-LAMP  （min） |
| --- | --- | --- |
| 1 | 35.3 | 19.0 |
| 2 | NA | 7.4 |
| 3 | 27.7 | 9.3 |
| 4 | 28.8 | 9.1 |
| 5 | 37.3 | 18.4 |
| 6 | 32.4 | 14.6 |
| 7 | 33.2 | 16.4 |
| 8 | 35.2 | 12.4 |
| 9 | 26.3 | 8.3 |
| 10 | 26.5 | 8.2 |
| 11 | 32.0 | 20.3 |
| 12 | 32.4 | 12.6 |
| 13 | 33.3 | 12.9 |
| 14 | 39.2 | 26.9 |
| 15 | NA | 6.7 |
| 16 | 35.5 | 25.4 |
| 17 | 33 | 28.6 |
| 18 | 30.4 | 15.6 |
| 19 | NA | 12.6 |
| 20 | 33 | 16.4 |
| 21* | 25 | failure |
| 22 | 32 | 27.1 |
| 23 | 24 | 9.1 |
| 24 | 32 | 39.8 |
| 25 | 31 | 13.0 |
| 26 | 33.2 | 11.9 |
| 27 | 30.0 | 10.9 |
| 28 | 32.2 | 11.4 |
| 29 | NA | 14.4 |

*, the sample was tested as negative by all 10 RT-LAMP assays. The possible reason is false positive by the RT-qPCR assay or wrong sample (negative samples was wrongly selected as positive sample).

NA， not available.

**Supplementary Figure S1. Genomic sequence alignments of seven human coronaviruses corresponding to six sets of selected primers.** A large gap in B3 of the Set-10 and the LF of Set-17 are shown by double slash (//). The GenBank accession numbers of the virus strains used here are SARS-CoV-2: MN908947.3, SARS-CoV: DQ182595.1, MERS-CoV: KM210278.1, HCoV-229E: NC_002645.1, HCoV-OC43: KF530068.1, HCoV-NL63: KU521535.1, and HCoV-HKU1: KF686344.1.
